# Response to Olaparib in a Patient with Germline *BRCA2* Mutation and Breast Cancer Leptomeningeal Carcinomatosis

**DOI:** 10.1038/s41523-019-0139-1

**Published:** 2019-11-29

**Authors:** Pedro Exman, Robert M. Mallery, Nancy U. Lin, Heather A. Parsons

**Affiliations:** 1 Medical Oncology, Dana-Farber Cancer Institute, Boston, MA USA; 2 Department of Neurology, Brigham and Women’s Hospital, Boston, MA USA

**Keywords:** Breast cancer, Breast cancer

## Abstract

Leptomeningeal carcinomatosis (LC) is a devastating complication of metastatic cancer that disproportionately affects patients with advanced breast cancer. Moreover, those with BRCA1/2-mutated disease more often experience leptomeningeal metastasis. Treatment options for LC are limited and often include significant toxicities. PARP inhibitors offer an important potential treatment for patients with BRCA1/2-mutated breast and ovarian cancers, but clinical studies excluded patients with central nervous system (CNS) metastases, including LC. Efficacy data in this area are therefore limited, although a phase I study of olaparib in glioblastoma did show CNS penetration. Here we report a case of a patient with BRCA2-mutated breast cancer and solitary recurrence in the leptomeninges with ongoing complete response to treatment with the PARP inhibitor olaparib. PARP inhibitors may be an important treatment option for patients with BRCA-mutated disease and LC, and warrant further study.

## Introduction

Leptomeningeal carcinomatosis (LC) is a complication of advanced cancer that carries a poor prognosis and most commonly affects patients with breast cancer.^1,2^ Indeed, in a retrospective case series of 103 consecutive unselected patients with LC from breast cancer, median overall survival was only 3.8 months, less than even the already poor estimates reported among selected patients enrolled in prospective randomized trials.^3^ More effective treatments for systemic metastatic disease have resulted in patients living longer, at an increased risk of LC.^4–8^ Yet, because of a dramatic paucity of data regarding the choice of therapeutic interventions, practice patterns vary widely.^9^ Pre-clinical or early phase I studies in the glioblastoma have demonstrated that poly-ADP ribose polymerase (PARP) inhibitors penetrate the central nervous system (CNS), suggesting a possible role of PARP inhibitors in the treatment of the brain or leptomeningeal metastasis.^10^ Breast cancer patients with hereditary *BRCA1/2* mutations develop CNS metastasis earlier than non-carrier patients, even when matched for age, stage, estrogen receptor (ER) expression, and human epidermal growth factor receptor 2 (HER2) expression, and are in need of effective therapies.^11^ We report a case of a patient with a hereditary *BRCA2* mutation and LC, who demonstrated an excellent clinical and radiographic response to olaparib.

## Case Report

A woman with a history of left-sided ductal carcinoma in situ diagnosed at age 48 years and who was treated with lumpectomy, radiation, and 5 years of tamoxifen presented with anatomic distortion in the left nipple 12 years later, at age 62 years. A bilateral diagnostic mammogram was initially unremarkable, but a mammogram 6 months later showed irregular nodules and architectural distortion in the retro-areolar region in the mid lower quadrant of the left breast. Further imaging demonstrated a 1.5 × 0.8 × 1.1 cm lesion and an ultrasound-guided core biopsy showed grade III invasive lobular carcinoma with significant perineural invasion. A left-sided skin sparing mastectomy with axillary lymph node dissection confirmed extensive grade II/III invasive lobular carcinoma measuring 10.4 cm and invading skeletal muscle. A 0.3 cm area of fibroadipose tissue in the deep surgical margin was positive for invasive carcinoma and metastatic carcinoma was present in 21 of 24 lymph nodes. Immunohistochemistry studies demonstrated 80% ER and 90% progesterone receptor positivity; HER2/NEU and E-cadherin were negative. The final pathologic American Joint Committee on Cancer (7th edition) staging was T3N3a. After the initial negative systemic staging, the patient received standard adjuvant chemotherapy with dose-dense doxorubicin and cyclophosphamide followed by paclitaxel starting 1 month after surgery, followed by postmastectomy and axillary radiation. She started on adjuvant endocrine therapy with anastrozole. Commercial genetic testing via Myriad Genetics, Inc., demonstrated a hereditary pathogenic *BRCA2* mutation (7558C > T). At age 62 years, she underwent risk-reducing bilateral salpingo-oophorectomy with pathology showing no evidence of ovarian carcinoma.

**Fig. 1:**
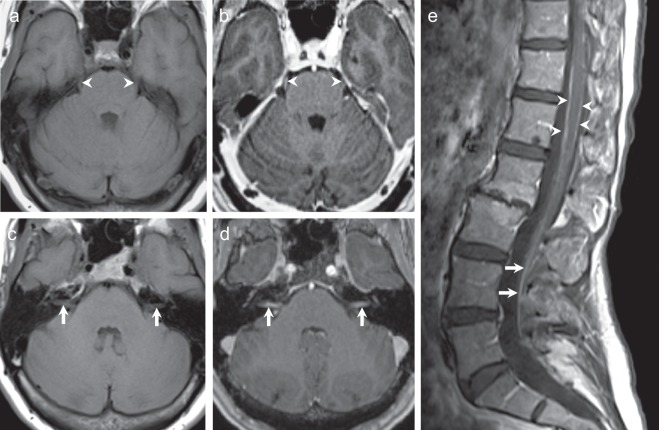
MRI of the brain and spine with and without contrast at diagnosis. **a**, **b** T1-weighted axial images show enhancement of the bilateral fifth cranial nerves after the administration of gadolinium contrast. **c**, **d** There is enhancement of the bilateral seventh–eighth cranial nerve complexes. **e** Sagittal T1-weighted post-contrast spine images demonstrate patchy circumferential enhancement along the thoracic and lumbar spinal cord (arrow heads) and enhancement of the cauda equina nerve roots (arrows), related to leptomeningeal carcinomatosis.

Eleven months after completing adjuvant treatment, the patient developed difficulty focusing her eyes, disequilibrium, and mild headache. Eastern Cooperative Oncology Group Performance Status was 1. Ocular motor examination by a neuro-ophthalmologist identified a small hypertropia of the right eye that increased in the left gaze, downward gaze, and with the right head tilt, consistent with a fourth nerve palsy. The remaining physical exam was unremarkable. Magnetic resonance imaging (MRI) of the brain and orbits with and without contrast showed enhancement of the bilateral fifth, seventh, and eight cranial nerves (Fig. 1a–d),^12^ and spinal MRI with and without contrast demonstrated patchy circumferential enhancement of the lower thoracic and lumbar spine, and enhancement of the cauda equina nerve roots, all consistent with LC. Lumbar puncture demonstrated an opening pressure of 24 cm cerebrospinal fluid, white blood cell count 2 (normal < 5), and protein 106 mg/dL (normal 19–40 mg/dL). Spinal fluid cytology was positive for malignant cells, confirming LC. Re-staging computed tomography (CT) scan of the chest, abdomen, and pelvis showed no evidence of other metastatic involvement.

**Fig. 2:**
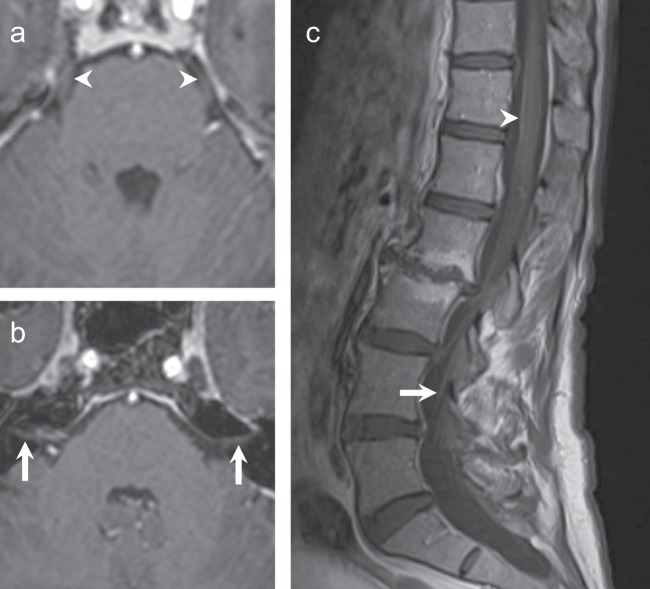
MRI brain and spine, 12 months following treatment with olaparib. **a**, **b** Axial T1-weighted post-contrast images show resolved enhancement of the fifth cranial nerves (**a**, arrowhead) and seventh–eighth cranial nerves complexes (**b**, arrows). Linear enhancement adjacent to the right seventh–eighth cranial nerve complex was consistent with a vascular loop. **c** Sagittal T1-weight MRI of the spine, post contrast, shows minimal enhancement anterior to the thoracic spinal cord (arrowhead) and subtle enhancement within the cauda equina nerve roots (arrow).

Anastrozole was discontinued on confirmation of metastatic disease. The patient provided written informed consent to participate in a Dana-Farber Cancer Institute Institutional Review Board-approved registry study including publication of this case report. Standard treatment approaches to LC were reviewed, including radiation therapy (RT) and intrathecal chemotherapy (IT-CT). Given her overall excellent performance status and low symptom burden, as well as the known limited efficacy and significant toxicity of standard approaches, we considered additional options. The patient started on olaparib 300 mg twice daily with good tolerance. After 4 weeks of olaparib therapy, her headaches, disequilibrium, and visual symptoms resolved. Repeat lumbar puncture was not performed given the strong correlation between clinical symptoms, physical exam, and MRI findings. No adverse effects related to olaparib were observed. The first re-staging brain MRI performed 4 months after starting olaparib demonstrated improved, mild enhancement of the cisternal segments of the cranial nerves VII and VIII, and no enhancement of the fifth cranial nerves. One year from LC diagnosis and initiation of olaparib, brain MRI showed complete resolution of previous cranial nerve enhancement (Fig. 2a, b). Spinal MRI at that time showed resolution of circumferential thoracic and lumbar spine enhancement, with only vague enhancement and no thickening of the cauda equina nerve roots (Fig. 2c). Body imaging continues to show no evidence of systemic metastatic disease. Nineteen months after initiation of olaparib for treatment of LC, she continues on the drug and remains asymptomatic with imaging showing ongoing response to therapy.

## Discussion

LC is increasingly common, comprising about 11–20% of CNS metastasis in breast cancer.^13^ Prospective studies have found a median overall survival of 9–30.3 weeks in patients diagnosed with meningeal metastasis.^3,14^ Hormonal status, HER2 amplification followed by trastuzumab treatment, and good initial performance status has been associated with a better prognosis in patients with breast cancer LC. In addition, some studies have demonstrated that cytologic and clinical response are both associated with improvement in survival.^15^ Data suggest that patients with *BRCA1/2* mutations and metastatic breast cancer develop CNS involvement, and that *BRCA2* mutation carriers develop CNS metastases at a higher frequency than non-carriers when controlling for subtype.^16^

Currently, there is no accepted standard treatment for patients with breast cancer who present with LC, and treatment options include RT, IT-CT, and systemic chemotherapy. RT may be more effective in patients with localized symptoms or bulky disease, and the application of focal radiation is preferred over craniospinal radiotherapy due to lower toxicity.^17^ Although IT-CT with methotrexate, cytarabine, or thiotepa has been considered the mainstay of the treatment for leptomeningeal disease, poor data are available to support its routine use. Four small prospective trials evaluated IT-CT and difference in the efficacy of these agents, and there was a discouraging median survival of 9–30 weeks and a significant rate of adverse events.^14,18–20^ IT trastuzumab in HER2-positive patients has demonstrated cytologic and radiographic responses in some case series, but its safety and efficacy remain under investigation.^21^

Recently, a shift toward the use of isolated systemic chemotherapy has gained strength. A small randomized trial comparing systemic or radiation treatment plus IT-CT with non-IT-CT treatment demonstrated superior neurological improvement in the non-IT-CT patient group and a numerical, but not statistically significant survival difference in the non-IT-CT group (18.3 weeks vs. 30.3 weeks for non-IT patients; 95% confidence interval (CI) −5.5 to +34.3; *p* = 0.32).^18^ In addition, patients who received IT-CT presented with more neurological complications (47% vs. 6%; *p* = 0.0072).^18^ Drugs with penetration across a disrupted blood–brain barrier (BBB), such as doxorubicin, capecitabine, and high-doses methotrexate may provide some clinical benefit. Lately, targeted therapies with small molecules, mostly in patients with driver gene mutation lung cancer, have been evaluated in patients with LC with mixed results.^22,23^ In this context, PARP inhibitors emerge as a possible alternative for the treatment of leptomeningeal disease in BRCA carrier patients.

Olaparib was the first PARP inhibitor approved by the US Food and Drug Administration for the treatment of metastatic breast cancer patients with deleterious germline alterations in BRCA1 or BRCA2. Pre-clinical data in animal models initially suggested a lack of CNS penetration of olaparib^24^ and early clinical trials excluded women with untreated CNS metastases.^24–26^ Subsequently, further work investigating other PARP inhibitors showed successful brain penetration in rodent models with disrupted BBBs due to CNS malignancy.^27,28^ An additional, recently presented study showed the limited brain penetration of another PARP inhibitor, rucaparib, in murine models with intact BBB. The authors showed that, despite this finding, antitumor effect was seen in a *BRCA1*-mutant intracranial murine model.^29^ These findings collectively suggest a potential role for PARP inhibitors in *BRCA*-mutated CNS malignancies, despite apparently limited drug penetration in intact BBB. A case study of a patient with *BRCA2-*mutated metastatic ovarian cancer involving the CNS provides clinical support for this approach.^30^

In the randomized phase III Olaparib Monotherapy Versus Physicians Choice Chemotherapy in the Treatment of Metastatic Breast Cancer Patients With Germline *BRCA1/2* Mutations (OlympiAD) study evaluating olaparib vs. treatment of physician’s choice in patients with metastatic breast cancer and a *BRCA* mutation, patients with treated, stable CNS metastases were eligible. A global progression-free survival (PFS) benefit was seen (hazard ratio (HR) 0.58; 95% CI 0.43–0.80, *p* < 0.001), and although this benefit was more pronounced in patients with measurable disease, no brain metastasis-specific analysis was performed.^31^ A phase I trial evaluating the use of olaparib in combination with temozolomide in patients with glioblastoma multiforme was presented recently at the 22nd Annual Meeting of the Society for Neuro-Oncology in 2017. The authors detected olaparib in 73 of 74 tumor core specimens from 27 patients and in 27 of 28 tumor margin specimens from 10 patients, indicating safety and drug penetration in patients with non-intact BBB.^10^

A second PARP inhibitor, talazoparib, has been recently approved for the treatment of germline *BRCA1/2*-associated metastatic breast cancer after the phase III EMBRACA trial (A Study Evaluating Talazoparib, a PARP Inhibitor, in Advanced and/or Metastatic Breast Cancer Patients with BRCA Mutation) compared talazoparib alone with standard chemotherapy in the first-fourth line treatment setting.^32^ A PFS benefit was observed (HR 0.54, 95% CI 0.41–0.71, *p* < 0.001) in the talazoparib arm when compared with chemotherapy of provider choice. Notably, 15% of patients in the PARP inhibitor arm had stable/treated CNS disease at baseline. In a subgroup analysis, these patients also demonstrated a PFS benefit compared with chemotherapy, with an HR to the left of patients without brain metastasis (HR 0.32, 95% CI 0.15–0.68 and HR 0.58, CI 95% 0.43–0.78, respectively), suggesting the possibility of a CNS effect of the drug.^32^ Unfortunately, similar to the OlympiAD trial, the EMBRACA trial did not allow patients with active parenchymal brain metastases or any history of leptomeningeal CNS metastases, and thus is unable to shed further light on whether PARP inhibitors are active in the CNS, with respect to CNS response rates or duration of CNS response.

Our patient with leptomeningeal metastasis in the setting of *BRCA2*-mutated breast cancer has maintained a complete clinical and a robust radiographic response to olaparib after 19 months of therapy. Although she has additional favorable prognostic factors such as an ER-positive receptor status, excellent performance status, and previously untreated metastatic disease, her response suggests efficacy of olaparib and her survival far exceeds reported medians for LC in breast cancer. In conclusion, the present case supports a potential role for PARP inhibitors in the treatment of LC and other CNS metastasis of breast cancer in patients with hereditary *BRCA* mutations. Given the relative paucity of patients with LC from *BRCA*-associated, metastatic breast cancer, a randomized trial to test PARP inhibitors vs. IT or other standard approaches will not be feasible. We believe that the exclusion of patients with active CNS disease from the early phase and registration trials of PARP inhibitors represents a missed opportunity, and we would encourage investigators to consider liberalizing the CNS inclusion criteria for trials testing PARP inhibitors in the future. Alternatively, registry or other approaches to harness data in the post-marketing setting may allow for a greater understanding of the CNS efficacy of PARP inhibitors and other novel agents.

### Reporting summary

Further information on research design is available in the Nature Research Reporting Summary linked to this article.

## Supplementary information

Reporting Summary Checklist

## Data Availability

Figs. 1 and 2 (MRI images) in the published article are the only data outputs of this study. Clinicopathological patient data used during this study are stored in a privacy-compliant locked database and cannot be made available to protect patient privacy. The data generated and analyzed during this study are described in the following metadata record: 10.6084/m9.figshare.10059926.^12^
